# An Undiagnosed Case of Chronic Pancreatitis With Multiple Visceral Arteries Pseudoaneurysm

**DOI:** 10.7759/cureus.14789

**Published:** 2021-05-01

**Authors:** Muhammad Naveed Anwar, Nouman Anthony, Qazi Kamran Amin, Zaland A Yousafzai, Hira Khalil

**Affiliations:** 1 Gastroenterology, Rehman Medical Institute, Peshawar, PAK; 2 General Medicine, Rehman Medical Institue, Peshawar, PAK; 3 General Medicine, Rehman Medical Institute, Peshawar, PAK; 4 Medicine, Rehman Medical Institute, Peshawar, PAK

**Keywords:** splanchnic pseudo aneurysms, celiac axis trifurcation pseudo aneurysm, gastro-duodenal artery pseudo aneurysm, pseudo aneurysm in jejunoileal branch of superior mesenteric artery, thrombosed pseudo aneurysm of superior pancreaticoduodenal artery, splanchnic artery pseudo aneurysm coiling and embolization, false aneurysm, peptide hydrolases, ethylene-vinyl alcohol copolymer, trisacryl gelatin microspheres

## Abstract

Visceral artery aneurysms, which could be either true or pseudo, are abnormal focal dilations of vessels supplying the abdominal organs. True aneurysms, by definition, suggest dilation of the vessel in response to increased blood flow, ultimately causing a blood-filled sac to form. Pseudoaneurysm, however, is the pooling of blood in surrounding tissues secondary to trauma or rupture. A 43-year-old woman G9 P9, known hypertensive was admitted electively for investigation of melena, hematemesis, hematochezia for one week along with weight loss and epigastric pain. Laboratory studies showed mild anemia with a hemoglobin level of 9.6 g/dL, hematocrit 29.5%, mean corpuscular hemoglobin (MCH) 26.7, upon which she was transfused two pints of blood and commenced at Injectable Vitamin K, injectable transamine, and infusion omeprazole. Two days later her levels improved to HB 12.4 g/dL, hematocrit 37.5%, MCH 26.7 pg, RBC 4.64 × 10*12/L. while being on treatment, a computed tomography (CT) mesenteric angiography was also conducted that showed multiple splanchnic pseudoaneurysms involving celiac axis trifurcation, gastroduodenal artery, superior/inferior pancreaticoduodenal artery, and jejunoileal branch of the superior mesenteric artery, and a large partially thrombosed pseudoaneurysm arising from superior pancreaticoduodenal branch causing significant mass effect on the second part of duodenum. On the basis of such findings, it was advised to perform coiling and embolization of the corresponding arteries. Multiple other small aneurysms with secondary arteriovenous malformations (AVM) were also seen. The whole circuit of flow retrograde and antegrade along with the aneurysm sac was blocked with multiple coils of variable sizes. An angiogram was repeated that revealed a good outcome. Pseudoaneurysms of the visceral arteries are very rare and affect mainly the splenic artery. The rarest of which is gastroduodenal artery (1.5%), pancreaticoduodenal artery (2%), and coeliac truck (4%). Therefore, this can be an incidental finding. The diagnosis is usually made with an angiography combined with clinical presentation. Variable treatment options are available depending on the patient’s fitness and hemodynamic stability. The endovascular approach, however, is mostly used in such cases.

## Introduction

Visceral artery aneurysms, which could be either true or pseudo, are abnormal focal dilations of vessels supplying the abdominal organs. True aneurysms, by definition, suggest dilation of the vessel in response to increased blood flow, ultimately causing a blood-filled sac to form. Pseudoaneurysm, however, is the pooling of blood in surrounding tissues secondary to trauma or rupture [1,2]. Pseudoaneurysms of the visceral arteries are exceedingly rare and affect mainly the splenic artery followed by the hepatic, superior mesenteric, coeliac trunk, gastric, intestinal, pancreaticoduodenal, gastroduodenal, and inferior mesenteric arteries [3], with rarest being those occurring the gastroduodenal (1.5%), pancreaticoduodenal (2%), and coeliac truck (4%) [4].

One possible etiology of pseudoaneurysms includes chronic pancreatitis, which causes leakage of proteolytic enzymes around the vessels that destroys the vessel wall. Erosion of a nearby pseudocyst into adjacent vessels, as well as blunt trauma to the vessel wall and postoperative complications that compromise vessel wall integrity, has also been suggested to cause pseudoaneurysms [5]. Pseudoaneurysms are diagnosed mainly through imaging and radiological findings combined with clinical presentation. In fact, digital angiography followed by computed tomography (CT) and ultrasound remains the gold standard for the diagnosis of pseudoaneurysms, with sensitivities of 100%, 67%, and 50%, respectively [6]. Given that pseudoaneurysms have a much higher risk for rupture compared to true aneurysms (76.3% vs. 3.1%), they are considered an emergency. Such conditions are extremely critical considering that gastroduodenal artery pseudoaneurysm rupture has a mortality rate of almost 40% to 70% [7].

Therefore, urgent treatment involving a surgical or endovascular approach depending on the aneurysm’s location, as well as improvement of the patient’s fitness and hemodynamic stability, is required [8].

## Case presentation

**Figure 1:**
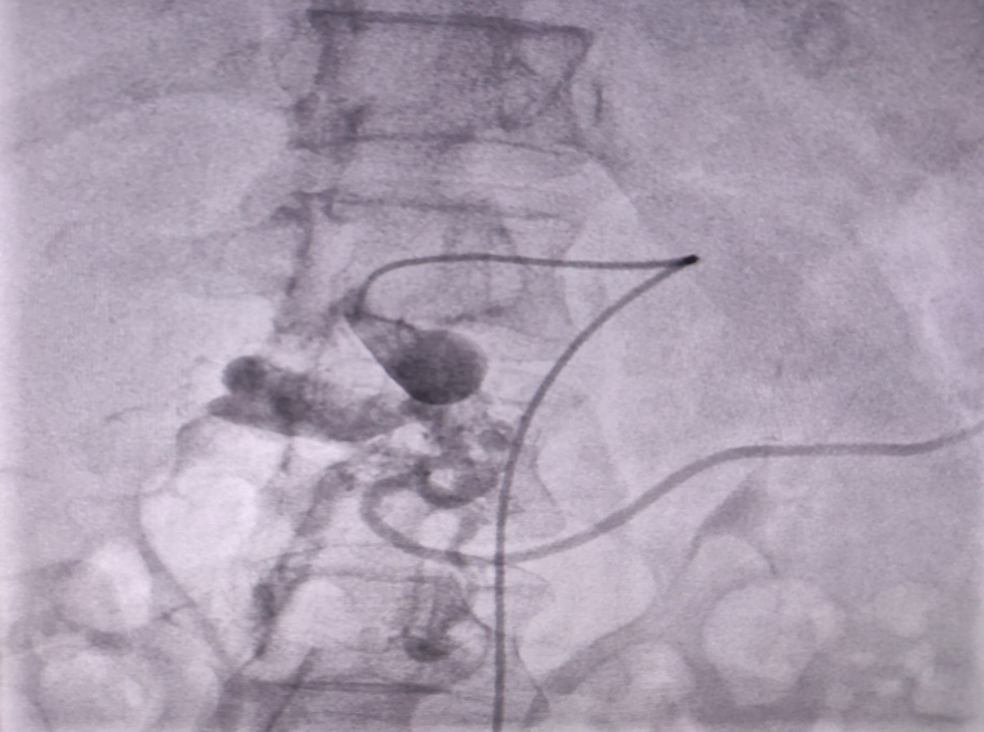
Pseudoaneurysms before getting coiled.

A 43-year-old female patient (para 9 with her last delivery two months prior) known hypertensive for 10 years presented to our department with gastrointestinal bleeding in the form of hematemesis, multiple episodes of melena, and hematochezia for one week. She also complained of weight loss and non-radiating and self-relieving epigastric pain not associated with positional changes. The patient was then assessed for causes of pseudoaneurysm formation, which primarily includes pancreatitis and cholecystitis. However, no significant abdominal tenderness, palpable abdominal mass, jaundice, flank tenderness, and increase in serum lipase and amylase were observed. The only significant findings included laboratory studies showing mild anemia with a hemoglobin of 9.6 g/dL, hematocrit of 29.5%, mean corpuscular hemoglobin (MCH) of 26.7, and red blood cells of 3.6 × 10^12/L, for which she was transfused two units of blood and started on injectable Vitamin K (10 mg, once daily), injectable tranexamic acid (500 mg, TDS), and omeprazole infusion (40 mg, once daily). After two days, her hemoglobin levels of 12.4 g/d, hematocrit of 37.5%, MCH of 26.7, and red blood cells 4.64 × 10^12/L. Meanwhile, mesenteric CT angiography also showed multiple splanchnic pseudoaneurysms involving the celiac axis trifurcation, gastroduodenal artery, superior/inferior pancreaticoduodenal artery, and jejunoileal branch of the superior mesenteric artery, and a large partially thrombosed pseudoaneurysm arising from superior pancreaticoduodenal branch causing a significant mass effect on the second part of duodenum as seen in Figure 1.

**Figure 2:**
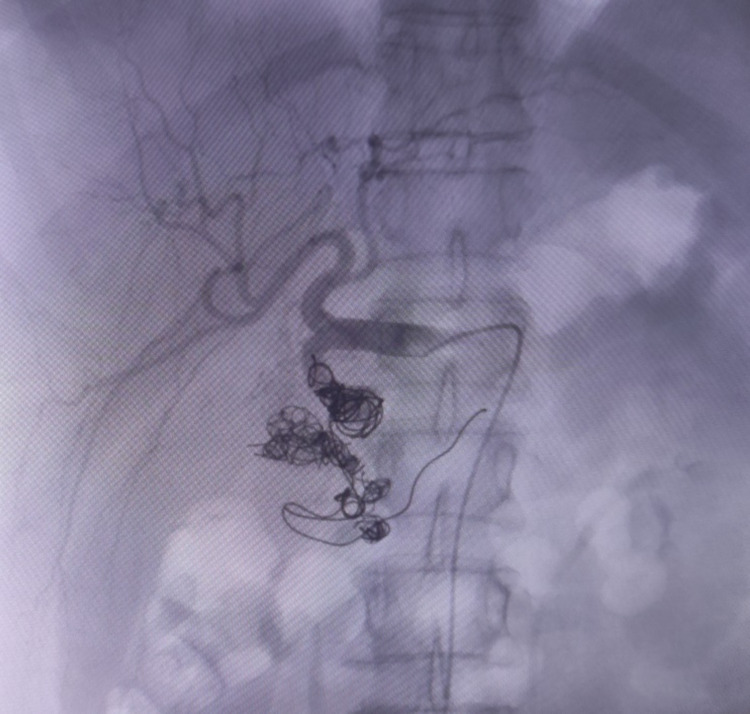
Initiation of coiling.

Based on such findings, coiling and embolization of the corresponding arteries were planed. Accordingly, the right common femoral artery was used as the access route with a retrograde 5-Fr catheter, which provided access to the celiac trunk and then into the gastroduodenal artery using a 4-Fr C2 and microcatheter. The large aneurysm in the gastroduodenal artery was identified and crossed. Multiple other small aneurysms with secondary aterio-venous malformations were also observed. The whole flow circuit (retrograde and anterograde) to the large aneurysm was packed with multiple coils, with the aneurysm sack also being coiled can be seen in Figure 2.

**Figure 3:**
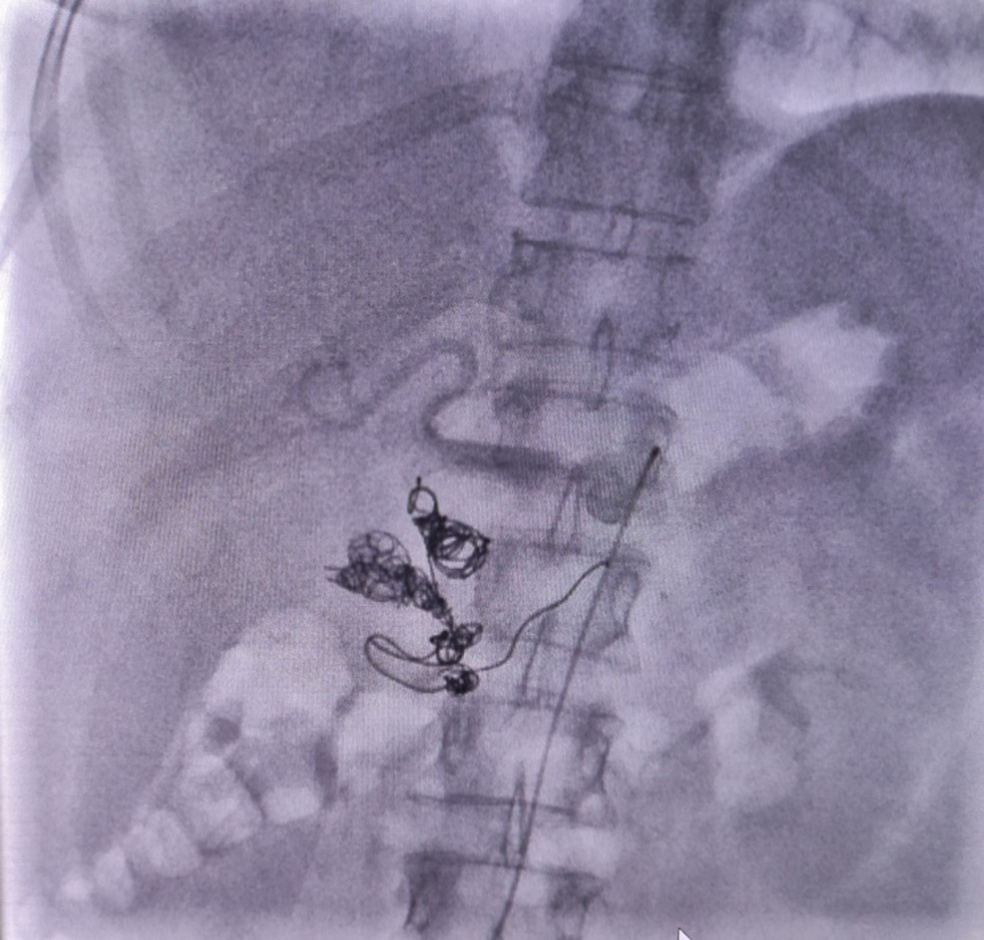
Coiling in progress.

The second aneurysm in the pancreaticoduodenal artery was accessed after multiple attempts, showing multiple coils of variable sizes blocking the aneurysm sack and its retro- and anterograde flow evident from Figure 3.

**Figure 4:**
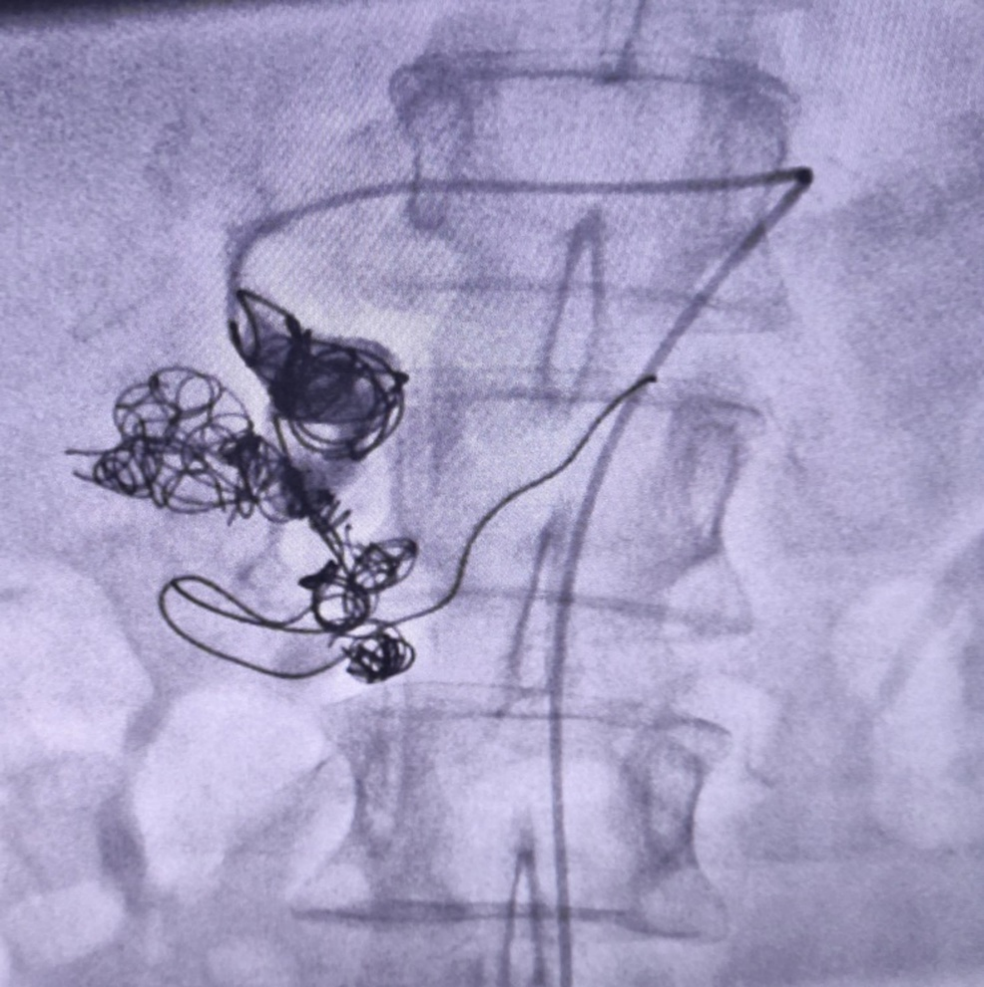
Shows completion of coiling.

As seen in Figure 4 repeat angiography revealed good outcomes, with no retrograde or anterograde filling of aneurysm and immediate complications having been observed.

Postoperative care included augmentin administration (1 mg, intravenous; two doses) with positional changes to prevent any hematoma formation. The patient was advised to keep her head tilted on 45-degree angle. She was observed for one more day for any hematoma formation or bleeding prior to discharge.

## Discussion

The present case showed multiple splanchnic pseudoaneurysms involving the celiac axis trifurcation, gastroduodenal artery, superior/inferior pancreaticoduodenal artery, and jejunoileal branch of superior mesenteric artery. Moreover, a large partially thrombosed pseudoaneurysm arising from superior pancreaticoduodenal branch had been found to cause a significant mass effect on the second part of the duodenum. The initial differential diagnosis established for this patient was chronic pancreatitis considering that it is the leading cause of pseudoaneurysm formation. Upon further investigation, she complained of unintentional weight loss and upper abdominal pain for the past few months, which she considered a consequence of her ongoing pregnancy.

The major vessel involved in pancreatic pseudoaneurysms is the splenic artery, which anatomically runs along the course of the pancreas before reaching the spleen. In cases where the pancreas becomes injured or inflamed, proteolytic enzymes that damage the corresponding arteries are released. As a consequence of autodigestion along with arterial wall weakening caused by the pancreatic juices, a focal pseudoaneurysm is formed. This explains why the splenic artery is the most common artery involved in pancreatic pseudoaneurysms [9]. Treatment options are dependent on the diameter and location of the aneurysm, as well as the patient's fitness and hemodynamic stability. True visceral aneurysm require treatment when its diameter is 2 cm greater than the respective normal artery, when rapid expansion occurs, (i.e., over >0.5 cm/year), or when the woman is pregnant or is of childbearing age [7]. Pseudoaneurysms, on the other hand, must be treated immediately given its much higher risk for rupture compared to true aneurysms (76.3% vs. 3.1%). Moreover, studies have shown that the rupture-associated mortality rate can range from 40% to 70% [7,10,11]. While treatment options include surgical techniques, such as arterial bypass, exclusion of the aneurysmal sac, and vessel ligature, less invasive endovascular techniques, such as coil embolization, stent placement, or a combination, have also been available. When the vessels supplying an end-organ are involved without any collaterals, the patency of the major vessel must be secured through stent placement or surgical revascularization. However, given that collaterals between the visceral arteries almost always exist, most pseudoaneurysms can be treated by embolization [11,12]. Considering that endovascular treatment is less invasive and can be performed under local anesthesia, it can be a good therapeutic strategy for patients who cannot undergo surgery due to severe comorbidities. Currently, the transcatheter selective embolization of pseudoaneurysms has become the most commonly used approach. Different materials, such as coils, gelatin foam, polyvinyl alcohol particles, trisacryl gelatin microspheres, Amplatzer vascular plugs, cyanoacrylate glue, and ethylene-vinyl alcohol copolymer (EVOH-Onyx®), can be used [4,12,13].

The Department of Vascular and Thoracic Surgery, Sint Vincentius Hospital, Belgium had reported two cases presenting signs and symptoms similar to that exhibited by our case (i.e., diffuse abdominal pain, vomiting, melena, and weight loss). Digital angiography in one of the aforementioned cases showed a complex pseudoaneurysm, for which stent placement with selective embolization of the afferent branches was performed. The other case presented with a non-complex pseudoaneurysm of the gastroduodenal artery that was successfully treated with coil embolization [14]. Another case involved a 39-year-old patient with alcoholic chronic pancreatitis who presented to the emergency department after a car accident showing a pseudoaneurysm of the gastroduodenal artery. The patient’s hemoperitoneum was treated with embolization of the gastroduodenal artery and pseudoaneurysm neck with coils [15]. Similarly, another case involved a 79-year-old man who presented with weight loss along with a 2-cm bleeding pseudoaneurysm in the gastroduodenal artery region, forming a large hematoma adjacent to the duodenum. Given that this patient was considered unfit for surgical repair given his abnormal fiver functions tests and increased serum amylase, coil embolization was performed with successful hemostasis [16].

Gastroduodenal artery aneurysms are indeed rare, accounting for only 1.5% of all visceral artery aneurysms. Such aneurysms can be detected incidentally or present with hemorrhagic shock, abdominal pain, and rarely with obstructive jaundice or hyperamylasemia, with its diagnosis usually requiring angiography. Recent evidence has shown that the endoscopic approach can be the new treatment option given its lesser invasiveness and better results as seen in the published case reports. However, further large-scale studies are needed to confirm such findings.

## Conclusions

Pseudoaneurysms quite rarely occur in the celiac trunk, superior and inferior pancreaticoduodenal, and gastroduodenal arteries. While mostly asymptomatic, asymptomatic splanchnic aneurysms have been increasingly diagnosed recently owing to modern imaging techniques, including CT, magnetic resonance imaging, and angiography. Although treatment options can vary according to the symptoms, the endovascular approach has recently gained popularity for asymptomatic splanchnic pseudoaneurysms.
